# Comfort Care Needs of Allogeneic Stem Cell Transplant Survivors: Lived Experience

**DOI:** 10.3390/healthcare12222217

**Published:** 2024-11-06

**Authors:** Lúcia Bacalhau, Patrícia Pontífice-Sousa

**Affiliations:** Institute of Health Sciences e Center for Interdisciplinary Health Research, Universidade Católica Portuguesa, Palma de Cima, Edifício Reitoria, 1649-023 Lisboa, Portugal; patriciaps@ucp.pt

**Keywords:** lived experience, phenomenology, comfort care, survivors, stem cells transplant, qualitative research

## Abstract

Introduction: Allogeneic Stem Cell Transplantation (ASCT) and, consequently, the chronicity associated with this life event have a growing prevalence and a significant impact on the life and daily life of each person who experiences it. It is necessary to reflect on the care needs of this group of vulnerable people. Comfort is a concern, and its improvement is a desired outcome of healthcare. To achieve this, it is necessary to know the needs of the people who are the focus of care. Aim: This paper aims to understand the needs of comfort care in the lived experience of comfort for survivors of ASCT. Methodology: Qualitative approach using van Manen’s phenomenology of practice. We uncovered the phenomenon through phenomenological interviews, which integrated narratives and illustrative episodes that reflected the lived experience of 20 survivors. Participants in the study were people who had undergone allogeneic hematopoietic progenitor cell transplantation, who were monitored on an outpatient basis and who met the following conditions: (i) were over 18 years old; (ii) had undergone SCT at least 3 months previously; (iii) had no evidence of disease relapse; (iv) were able to express themselves verbally, providing information relevant to the study, as well as expressing emotions and feelings. Descriptions of lived experiences were collected from participants between July 2020 and May 2021. In the phenomenological reflection on the lived descriptions, we followed the “stages” epoché, reduction, and vocative. Results: The following themes emerged from the ASCT survivor’s lived experience of comfort related to comfort care needs: continuous follow-up, reference nurse, information, assistance in adapting to the new self, mental health intervention, spiritual support, adaptation to changes in sexuality, physical rehabilitation, and job reintegration. Conclusion: This study reveals the importance of continuous follow-up for ASCT survivors. Survivors experience major changes in their lives in the long term and require a response from health professionals to find comfort in their daily lives.

## 1. Introduction

The process of living with an illness gives rise to internal and external experiences, which are experienced in a unique way, elaborated in the light of each person’s personal experience. The search for answers during a situation of illness, as in the case of a survivor of ASCT, with all the physical, psychological, social, spiritual, and existential implications that this entails, takes on its own desirable meaning.

In people with cancer, awareness can be influenced by the acceptance of the state of health and the side effects of the treatments carried out. It is, therefore, necessary for these people to reflect on and accept their state of health and to integrate the changes and the process of managing the disease and the side effects resulting from the treatments [1].

People who have undergone stem cell transplants are living through a complex process that brings with it multidimensional and specific sensations, emotions, thoughts, and experiences. They are people who challenge healthcare due to their specific and long-term care needs. The awareness that there is a new group of people, cancer survivors, with specific needs related to sequelae or complications resulting from the treatments they have undergone, is a reality [2,3]. The early detection of cancer and increasingly effective treatments have changed the course of the disease, leading to a growing number of people surviving cancer and living beyond cancer for many years to come [4]. Knowing that each person tends to experience the situation and circumstance individually, we adopted the concept of survivor defined by Crystal and his collaborators [5] when considering the person who has completed treatment for their disease and is in remission.

A greater number of hospitalizations were recorded among HCT survivors than among non-HCT cancer survivors. Furthermore, they were at a greater risk of experiencing all categories of late outcomes in comparison to the general population. HCT survivors exhibited a higher incidence of infections and respiratory complications, both infectious and non-infectious, in comparison to non-HCT cancer survivors [6].

In many clinical situations, cancer has gone beyond the death sentence and has become part of the group of chronic diseases [7]. It has become a condition characterized by uncertainty, with long-term side effects resulting from the disease and/or treatments to which psychosocial problems are added [8].

Cancer survivors are growing, and a new intervention is needed to meet their needs [9,10]. Cancer interferes with all areas of the quality of life and the consequences of treatments, which can arise or persist for years, often have an impact on the well-being of survivors [11,12]. The increase in the survival of these people makes us aware of the challenge set by the World Health Organization: it is not enough to add years to life, but it is crucial to add life to years, so that we can go beyond the exclusively technological and biomedical aspect, moving towards the humanization of care and investing in people’s quality of life [11].

The Institute of Medicine’s report From Cancer Patient to Cancer Survivor: Lost in Transition aimed to raise awareness among healthcare professionals about the issues faced by cancer survivors to improve their quality of life (QOL) [13,14]. This report led to the implementation of survivor care plans in several institutions in the USA, and some studies and reflections on their application have emerged [2,15,16,17,18,19,20].

Reflecting on the comfort of these people implies understanding their lived experience. The word comfort comes from the Latin, *confortare*, and means to restore physical strength, vigor, and energy; to make strong, to strengthen, to invigorate and to give encouragement. We have found that there are several definitions of comfort that appear in the literature in accordance with the perspective of each author. Although several studies have emerged on the concept of comfort, this definition remains complex. Comfort is considered a noun, verb, adjective, and, simultaneously, a state, a process, and a result [11]. Although complex for many authors but essential in holistic human care, it is a central concept in the discipline of nursing and presents itself as an integral part of the care process, being the most important dimension of it.

The concept of comfort is regarded as a significant and highly sought-after quality that is perceived as valuable throughout the lifespan. As a subjective state, it can be defined as a person’s experience of feeling benefited, empowered, and enabled. This translates into a pleasant sensation, even though it may be transient, which is expressed in a positive manner. It is an experience of personal empowerment, with different meanings for different individuals [11].

The concept of comfort care is predicated on the notion of meeting the individual’s needs and developing a constant concern for their overall well-being. As a comprehensive model of care based on the principles of humanity and beneficence, it is designed to advance the person’s best interests and enhance their quality of life. Consequently, a comforting intervention addresses a multitude of variables and is conducted through interaction, which may be defined as an “encounter/involvement” in a co-constructed action with the individual receiving care. A prerequisite for this is an understanding of the individual’s experiences, which enables the comforting action to be perceived as an attitude of commitment that meets the person’s needs and particular circumstances [11].

In evaluating comfort care, it is evident that the situational context must be considered in addition to addressing the individual’s needs. A crucial aspect is the identification of discomfort to gain insight into the nature of comfort [12].

The focus of this study is on people who have experienced a transition from illness to survivors of SCT and are experiencing chronic illness. Reflecting on the survivor’s lived experience of comfort, as well as all the uniqueness and complexity of the inherent problems, is fundamental for the development of care practice in this context.

The aim of this study was to investigate the care needs in the lived experience of comfort of SCT survivors.

## 2. Materials and Methods

### 2.1. Design

A qualitative research approach was used in this study. We used van Manen’s phenomenology of practice as a research method. In an invitation to “openness” to the experience of lived meaning, in the search for a new way of thinking, the phenomenology of practice constantly looks for renewing and creative aspects of lived experience and meaning [21].

### 2.2. Participants

The participants in the study were individuals who had undergone allogeneic hematopoietic progenitor cell transplantation and were monitored on an outpatient basis. They met the following conditions: (i) were over 18 years of age; (ii) had undergone SCT at least three months previously; (iii) exhibited no evidence of disease relapse; (iv) demonstrated the capacity to express themselves verbally, providing information pertinent to the study, as well as expressing emotions and feelings.

The selection of survivors was based on a number of factors, including age, gender, clinical diagnosis, family presence, the presence or absence of graft versus host disease (GVHD), and the time elapsed since stem cell transplantation. This approach was taken in order to ensure the representativeness and relevance of each person’s experience in understanding the phenomenon under study [21]. In order to facilitate the comprehension of the experience, consideration was given to the availability and willingness to share the lived experience [22,23]. This approach was designed to guarantee the relevance of the participants’ experiences and expressions [23].

Study settings and recruitment of participants: The selection of survivors was based on a number of factors, including age, gender, clinical diagnosis, family presence, the presence or absence of graft versus host disease (GVHD), and the time elapsed since stem cell transplantation. This approach was employed to ensure the representativeness and relevance of the sample. This approach allows for the understanding of the phenomenon under study, as proposed by van Manen [21]. In order to facilitate the comprehension of the experience, consideration was given to the availability and willingness to share the lived experience [21,23]. This approach was designed to ensure the relevance of the participants’ experiences and expressions [23]. Contact was made with the survivor in person at the outpatient unit of the Bone Marrow Transplantation Unit in Portugal. The objective of the study and the methodology used were explained to him/her, and, depending on his/her interest, acceptance, and availability, an interview was scheduled. In addition to the interview, the participants volunteered to share poems and texts written by themselves, which were descriptions of their lived experience and significantly illustrated their experience.

It was agreed when the interview would take place and how the participants would be able to provide experiential material. As the data collection took place during a pandemic, it was important to find a comfortable and safe place. The interview took place in a private and quiet area of the health center, ensuring the place was ventilated, a safe distance was maintained, personal protective equipment and equipment was worn, and the researcher had taken a Covid test. We tried to ensure the safest possible environment so that the person felt comfortable sharing their experience. This new reality requires reflection on the risks to the survivor and how personal equipment, particularly the mask, interferes with the development of the phenomenological interview interaction.

### 2.3. Data Collection

Data were collected online between July 2020 and May 2021. The instrument used to collect data was a phenomenological interview. Following the guidelines of van Manen [21] the subjects of the study were asked to focus on a particular experience, describing the event specifically, leading the survivor to highlight what they experienced, as if they were going back to the moment. Using language that was easy to understand, we guided the collection of these descriptions in the interviews, making sure that the description was specific in what they felt and what they experienced. Illustrative material was also collected from sources such as literature, in this case written by the survivors themselves, chosen by the participants who served as catalysts for reflection, to illustrate and expose aspects that better elucidated the phenomenon, clarifying it [21]. A means of communication with the researcher was also made available in case the survivor had any relevant aspects to share.

Twenty interviews, with an average duration of 120 min, were conducted. The interviews were audio-recorded and transcribed verbatim. Participants were given the chance to check the content of their transcriptions, and the recrafted stories derived from them. This way, participants could validate their contributions and the meaning of their experiences. They all confirmed that the stories were documented in an appropriate manner.

In van Manen’s hermeneutic phenomenological approach, the collection of empirical material ends when a deep and complete understanding of the lived experience of the phenomenon under study has been achieved. This point is not measured by the number of participants or the amount of data but by the depth with which the meaning and essence of the experience is understood [21].

The collection of experiential material ended when the themes appeared repeatedly in the participants’ descriptions, indicating that the experiences were sufficiently represented. This suggests that the data provided a rich and dense understanding of the phenomenon. We found that new perspectives on the lived experience had been exhausted, meaning that several dimensions and variations of the experience had already been collected. The collection has produced sufficiently rich experiential examples and descriptions to illustrate in depth the central meanings of the phenomenon. Van Manen suggests that phenomenological interpretation is a continuous process of writing and rewriting until the essence is captured. When data analysis allows themes to be identified with significant coherence and depth, this is a sign that the phenomenon has been understood and the collection of experiential material can be completed [21]. Consequently, the compilation of experiential material was completed with 20 participants.

### 2.4. Data Analysis

Phenomenological research is not identified with a set of sequenced procedures carried out in isolation. It assumes the need to carry out certain steps in a structured order, such as collecting experiences for later analysis. In this order of ideas, we followed the steps that van Manen) presents as “stages” [21]: the epoché or suspension, the reduction, and the vocative, which were procedures that we adopted during the analysis of the collected material.

First, after transcribing and compiling all the experiential material, we proceeded to (i) read the whole, holistically, to grasp the meaning and sense of the whole [22,24], (ii) We then identified the essential phrases of the lived experience under study, and, (iii) in a third step, we highlighted the thematic units extracted from the descriptions that fall within the essential themes related to the phenomenon. At this stage, we looked at all the sentences and groups of sentences and were guided by the question: what do these sentences or groups of sentences reveal about the lived experience described? We used Maxqda software 2020 to support the construction of themes from the experiential material collected.

### 2.5. Rigor and Reflexivity

To ensure the quality and trustworthiness of the research, for the qualitative paradigm, we met the following criteria adapted to the phenomenological context of the study according to van Manen recommendations credibility, i.e., the ability of the participants to confirm that the constructed text is true to what was lived in their comfort experience, where the richness of the phenomenological texts provide the trustworthiness and confirmability [21]. In order to meet these requirements, the following specific strategies were followed, emphasizing the phenomenological nature of the study: (i) reflection with prolonged engagement, which was considered necessary to answer the research questions, to capture the culture of the participants and to identify conflicting information introduced by the two researchers’ distortions, ensuring that epoch and reduction were achieved; (ii) persistent observation of descriptions of lived experience; (iii) audits of the research and writing process undertaken, where external readers with knowledge of the method were allowed to check the reflective process undertaken to validate the researcher’s thinking, the key decisions made, and the way in which the lived-experience descriptions was collected and reflected upon and written up, such as the conclusions reached; (iv) and participant review, where the results of the reflection undertaken were returned to the study participants, and the meaning of their lived experience was verified by them. The construction of the final text of the phenomenological reflection on the lived-experience descriptions was carried out only after the approval of the whole process [24]. These steps were relevant, given the inexperience of the researcher, and the sharing, reflection, and discussion of these aspects with other researchers benefited the conduct, construction, and writing of the phenomenological reflection on the lived-experience descriptions.

### 2.6. Ethical Considerations

Informed consent was obtained from all participants before commencing interviews. The ethical standards established by the Declaration of Helsinki were followed, and personal data were processed following Regulation (EU) 2016/679 of the European Parliament and of the Council of 27 April 2016, on the protection of natural persons concerning the processing of personal data and the free movement of such data, and repealing Directive 95/46/EC. The study received approval from the Research Ethics Committee where it was conducted (UIC/1314).

## 3. Results

The participants in this study are heterogeneous. They illustrate some of the characteristics of ASCT survivors. The specific characteristics of the participants can be analyzed in Table 1, Table 2 and Table 3.

**Figure 1:**
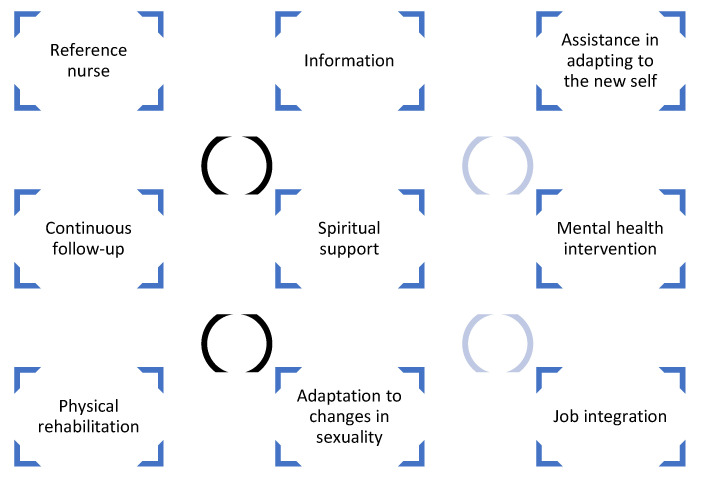
Sub-themes in the “Comfort care needs” theme.

Needs emerge in the experience of comfort as a theme and are recognized by the study participants as areas and forms of intervention that provide the experience of comfort. Survivors report needs that last over time and identify nurses as the professionals with the knowledge and skills to respond in a comforting way to these needs. Figure 1 illustrates the organization of the sub-themes in the “Comfort care needs” theme.

### 3.1. Continuous Follow-Up

It is suggested that a telephone follow-up structure be set up for the survivor to understand what the person is going through and the intervention needs of health professionals. João and Matilde recognize that telephone monitoring allows them to maintain contact with the hospital and avoid travelling to a place that causes them discomfort. This continuous contact allows them to feel protected and safe.

João: “*I think that in the first year and the second year, if we are accompanied, even with a phone call, because we have more openness to talk to you (nurses). I think that with you (nurses) we feel comfort, I think that this phone call makes sense and helps us.*”

Matilde: “*If I have regular contact, I feel I’m being accompanied, I clarify doubts, I commit to doing activities. The phone call allows me to contact you from a distance, I don’t have to travel to hospital and I feel protected, accompanied, it gives me security.*”

It was also suggested that help groups should be set up. These promote the sharing of experiences, the recognition that the experiences of other survivors are similar, and stimulate adaptation to the new time of life and the inherent circumstances. With the support of the nurses, the survivors meet to share their life experiences and to find ways to help each other like Duarte and João said:

Duarte: “*I think there should be brainstorming, get a bunch of survivors together, 5 or 6 or 7, gather in a space and talk to each other and then you can make comparisons, studies.*”

João: “*I need to share what I’m feeling with people who have lived through it, to learn about other people’s experiences in order to understand what’s normal and what’s not. Only by listening to other people’s experiences will I be able to understand some aspects of what I’m going through.*”

We have identified the need for prolonged accompaniment and peer-to-peer sharing in order to find the sense, meaning and understanding of what is being experienced.

### 3.2. Reference Nurse

It was felt that there was a need for a reference nurse in the process of experiencing the improvement of comfort, to guide the person in building the relationship, and to guarantee a deeper understanding of the survivor and the development of trust with them.

Anita: “*I think your support is always very important.*”

Camila: “*We have doubts about what we can eat and when we can start eating. We could have this support with you. This family nurse would be closer to the family and would be an asset to them, not just in clinical terms.*”

### 3.3. Information

The provision of information by nurses with experience in transplantation is important for the survivor. It is assumed that many doubts arise during the survivor’s experience and that nurses are an important source of the clarification, advice, and promotion of healthy lifestyles compatible with the immunosuppression experienced:

Amélia: “*I think it makes sense; I think there are solutions to some of the problems we have that we don’t know about. We try to comply with what we’re told, but there may be solutions that you know of for the problems we have.*”

Anita: “*One of the things that could be improved is control over vaccinations. I was one of the first in my group to be vaccinated. (...) More information about this, or even a website where these doubts could be clarified.*”

Camila: “*It’s like when I ask, “Can I take a bus?” That’s the kind of question I could ask a nurse, instead of a doctor, and then I wouldn’t have to spend time at the doctor’s appointment.*”

Francisco: “*Yes, it’s necessary because there are many doubts. There is a need for information.*”

### 3.4. Assistance in Adapting to the New Self

There is a need for support to adjust to the new reality and new normal, where the need for information arises in various areas, and to adapt to limitations or changes to their new surviving self. The need for information about eating during the period of neutropenia and immunosuppression (what foods to eat, how to cook them, what cannot be eaten, and when restrictions can be eased) is an aspect that is mentioned by survivors. They feel that nurses have an important place in this area of knowledge about the transplant process:

Afonso: “*There is a need for information. For example, about food, about social support, about food (…)*”

Camila: “*A bad diet, whatever it may be, can cause a person to be hospitalized with some kind of problem that will cost the state a lot more than if we had a nurse calling to ask how they are, if they’re on a diet or something.*”

### 3.5. Mental Health Intervention

The need for nursing intervention in mental health is relevant to the survivor. This is felt to be a fundamental area where greater investment and development is needed. Participants feel that once medical treatment is over, nursing intervention ends, although the need for mental health support becomes necessary in continuity due to everything that has been experienced and the changes that have arisen in the various domains of the survivor’s being, so that life acquires a comforting purpose:

Amélia: “*Even in terms of psychological support, I think it’s sometimes important. There are many ghosts that live with us.*”

Diogo: “*I’ve always thought it’s important to provide support. Not all of us have the capacity to overcome or suffer from this type of illness or problem, so I always think it’s important to provide mental health support.*”

### 3.6. Spiritual Support

The need for spiritual support is felt to be the nurse’s domain. There is a feeling that what is needed transcends the physical body and extends into the spiritual realm. These changes accompany the person for the long term. The need for support in this area is felt:

Afonso: “*It would be good to talk about this, about the spiritual part, and to be able to take advantage of the experience of the nurses who accompany the patients for longer periods of time to talk about it and understand to what extent it is normal or to be expected. I think that yes, there was room for this kind of intervention, I’m not sure in what form, but I think it would be an interesting form of support, different from psychological support, which is more technical, and in the case of psychiatrists it’s more drugs, and not everything can be solved with drugs!*”

Matilde: “*There is a component that is being neglected, there is the physical part, there is the psychiatric part, but there is a lack of spiritual support. And this part that I’m talking about, which could be explored, there are volunteers from the league, for example. The physical part is guaranteed, the psychological part too, but the spiritual part is not. It could be the comfort of a word, a conversation, but it must be someone who knows what they’re talking about. It’s not like someone comes and asks you how you’re feeling, and you don’t even hear the answer, it can’t be like that.*”

### 3.7. Adaptation to Changes in Sexuality

Sexuality is perceived by survivors as an area for intervention. Availability, space and time are needed for the person to ask questions and clarify problems and changes in sexual activity. The participants in the study identified nurses as the professionals who have the ability to promote satisfaction with sexuality in a close and individualized way.

Camila: “*The question of exercise, diet and even sex. Sometimes people have doubts and are ashamed of what they are asking.*”

Matilde: “*Then there is another part that I think also deserves different support: our sex life, our intimate life, isn’t talked about and I think that’s important. I think it is important to address the issue of diet and sex life, you nurses could do it.*”

### 3.8. Physical Rehabilitation

Concern about physical rehabilitation is important for the process of regaining comfort in everyday life. There are doubts about the possibility of mobilizing the body, the exercises that can be performed, and the abilities that the survivor can recover. The encouragement of the nursing professional is also valued as important in this area of the survivor’s life:

Camila: “*Nurses could be an asset in nutrition and sports. They could make a physical exercise plan with us, even if it’s just to go for a 15-min walk this week and then, even if it’s just to make a phone call, it’s not even worth it for the person to go, to find out if it went well or not, then the person feels obliged to do it because there’s someone on the other end who’s going to ask them about it.*”

Francisco: “*There is a need for information on exercises that we can do in this situation so that we don’t lose even more capacity.*”

### 3.9. Job Reintegration

The nurse appears in the comfort experience as the professional who can contribute to the survivor’s social reintegration into the world of work, on the one hand, because they can promote the survivor’s training to resume work activities and, on the other hand, because they can promote the preparation of employing institutions to create conditions to reintegrate the survivor into the work context:

Miguel: “*It’s a time when I think more specific guidance would help with the reintegration process, because there’s a stigma, we’re afraid, there’s a stigma too, it’s not something you tell people you’ve just been through, nobody deals with it normally. People end up being afraid to deal with you. (…).*”

Santiago: “*(…) the employer doesn’t understand what we’ve been through or what we still feel. It would be important for them to be aware of what we’ve been through and the after-effects we’ve suffered, so that they could understand our side of the story.!*”

## 4. Discussion

The survivors mentioned the need for continuous follow-up with nursing support and understand that the nurses in the SCT unit have knowledge about the process they are going through and that this can be used productively to build a path of comfort during the survival experience so that they can find a sense of purpose in their lives and adjust to the new normal. In a similar vein, Grant and their collaborators [25]) also defended the necessity of nursing care following discharge from the SCT unit, given that these individuals are particularly susceptible to infection and readmission to hospital. As Cooke [26]) have previously observed, it is of the utmost importance to prevent and detect complications at an early stage, thereby reducing readmission rates and providing the individual with the information they require to adapt to the physical, psychological, social, and spiritual processes involved in transplantation.

Likewise, the way in which this support is provided is also thought of by the survivors insofar as they value the telephone support provided by the unit, although they point out that this could be developed in a structured way, with regular contact with the survivor on a regular basis, according to their needs and for a long time after the transplant. Dyer and their collaborators [27] advocated for the use of this strategy of maintaining contact, and Amonoo and their collaborators [28] referred to the importance of this type of follow-up for improving the quality of life of the survivors in their study.

The importance of survivor group discussion about aspects of transplantation was mentioned as important by one of the survivors, considering that sharing experiences allows for the clarification of certain aspects of the experience and fosters mutual help, communication, and the sharing of knowledge. The study by Sekse and their collaborators [29] showed that participation in the rehabilitation group was described as a special community of mutual understanding and belonging. Education and the sharing of knowledge provided a broader understanding of the women’s experiences. The presence of dedicated workers and care professionals was reported as being essential to the group’s external intervention. The program also provided professionals with important insights into people’s views and feelings about cancer treatment, trajectories, and rehabilitation. This type of knowledge sharing improves communication skills and psychosocial support related to gynecological cancer care [28]. It is necessary to innovate and guarantee close care. The role of the reference nurse is necessary for the continuity of care for the participants in our study. The nurse reference method promotes the co-reponsibility and involvement of nurses, is a guarantee of quality care, stimulates the nurse–person relationship, favors the coordination and evaluation of care, promotes the use of the nursing process, and is even essential for the implementation of this method. The findings of this study align with the principles of the nurse reference method, which emphasizes the importance of personalized care for the individual and their family. This approach strengthens the relationship between care partners, facilitates the planning and evaluation of care, and enables communication that is based on truth, trust, and mutual respect [30].

In terms of mental health, survivors say that they feel the need for prolonged support from people who can understand the reality of the person who has undergone a transplant. They feel as if this area of intervention is being neglected, even though it is one of the areas that causes the most discomfort during survival, and this is relevant and stands out from other studies.

Spirituality is a focus of attention for survivors and emerges from their daily lives as an aspect that needs attention from professionals as a way of promoting and guaranteeing well-being based on comfort. In fact, spirituality requires a complex reflection that involves understanding it and how it can be affected by uncomfortable states to better meet the spiritual needs of the people being cared for [31]. Spiritual support helps protect against death anxiety and worries about loss of dignity, as well as helping to cope with the reality of imminent death [32].

For survivors, it feels necessary to adjust to the new normal, integrating information and adapting to the new survivor self. In this logic, in relation to food, survivors refer to the restrictions associated with the neutropenic diet that they must comply with during the post-transplant period; this is important and different from other studies. It is up to nurses with skills in SCT to incorporate this knowledge of the neutropenic diet and to coordinate with the medical team the necessary adjustments for the best survival of these people [33].

Sexuality is another area that survivors feel needs to be addressed and assessed, with coping strategies defined, comfort improved, and sexual health promoted. Like in other studies [34,35,36,37] showed that changes in the experience of sexuality are quite common and interfere with these people’s experience of life. Mental health interventions should be combined with the area of sexuality to improve the quality of life after the illness, including a component on sexual well-being, and interventions in sexuality should incorporate components on psychological and relational functioning [36,37].

The rehabilitation of physical abilities is felt to be necessary. They felt the loss of muscle mass, fatigue, functional alterations in the organs affected by GVHD, the time of decreased physical activity, and even the processes of anxiety and depression experienced. The literature argues that attention should be paid both to the direct effects of the inflammatory process of chronic GVHD and to the side effects of the drugs used to treat the disease. Developing intervention in this area involves promoting the recovery of abilities to improve comfort. More research is needed into rehabilitation interventions related to physiological outcomes, involving members of the multidisciplinary rehabilitation team [38,39,40]. According to the recommendations of the International Classification of Functioning, Disability and Health, a combined assessment helps health professionals and stakeholders to understand a patient’s unique challenges and strengths and thus design an individualized therapeutic approach [40].

Integration into professional activity is also mentioned by survivors as a necessary area of intervention by nursing professionals, who facilitate the recognition of skills, places, and forms of safe work, as well as clarifying the real situation of survivors to employers, taking into account their abilities and limitations. Stepanikova and their collaborators [41] found that survivors described a variety of challenges in these areas, including job insecurity, discrimination, career derailment, the lack of career direction, delayed goals, financial losses, insurance difficulties, constraints on job mobility, and physical/mental limitations. The results provide a meaningful interpretation of the findings.

The participants expressed feelings of discomfort associated with a sense of futility regarding the time they currently had available. The promotion of activities that are aligned with each individual’s functional capabilities can facilitate an enhanced perception of their quality of life, a strengthened sense of purpose, and an increased resilience to life’s challenges. Conversely, some employers remain uncertain about the capabilities of individuals who are managing these conditions. Recognizing that these individuals may require alternative strategies to facilitate their employment can foster a more comfortable environment for both the employer and the employee. These professionals will be able to build multidisciplinary teams that are concerned with the professional and social integration of these people so that the feeling that they are alive and productive is maintained. In one review of the literature [42], had highlighted the need to build rehabilitation programs in which insertion into the workplace is introduced, looking for alternatives according to the individuality of each subject, which promotes the rehabilitation of physical functions and psychological adjustment to the current health situation, promoting well-being and the quality of life.

The literature shows the need to develop combined interventions in the various areas addressed by survivors in ongoing multidisciplinary rehabilitation and follow-up programs for survivors where nurses have a fundamental role in intervening and ensuring that the programs are adapted to the individuality of each survivor [39]. The main purpose of these programs is to improve the survivor’s lived experience, ensure adaptation by providing coping strategies for the new being that has emerged from the experience, and promote functional capacities to recover or reorganize life roles.

### Limitations

The main limitation of this study was that it was carried out in just one Stem Cells Transplant Unit. We consider it important to replicate this study in other units to gain a more comprehensive understanding of this experience. Due to the phenomenological nature of this study, its findings cannot be extrapolated to other realities. We would point out that this study was carried out during the COVID-19 pandemic, which may have influenced the participants’ lived experience of comfort.

This study suggests the importance of further scientific research with the development of a longitudinal study with this population with several evaluation moments. Other studies we suggest are looking at the construction of knowledge around stem cell transplantation, what processes are inherent in the construction of coping processes during survival from stem cell transplantation, understanding the lived experience of comfort during the transplantation process, and understanding the influence of nursing intervention on the experience of comfort in isolation units.

## 5. Conclusions

We understand that the meaning of comfort is defined by feelings and emotions and is influenced by expectations, desires, and the time of occurrence of each feeling. To adjust to their new world, survivors establish energetic relationships with their surroundings in an active and reflective way, being able to identify the desired health standards and contribute to improving and adjusting the professional practices of the contexts in which they move.

The conditions that determine the experience of comfort are centered on specific domains of each person, their construction, their perception of themselves and others, and their desire to achieve more comfort. This sense of seeking more and better comfort, within what is experienced, emerges from what is shared by each survivor. It differs from other studies in that the need for comfort care is focused on the individual recovery process, without mentioning family members or other elements of the social nucleus. This is justified by the need for individual recovery to promote capacities and make them capable and autonomous in the different areas of life.

In the experience of comfort, nurses appear as the professionals to whom comforting answers are sought. The different areas of competence of these professionals are understood, recognizing the multiple areas of intervention. In addition to the reference nurse, it is intended that nurses who are experts in specific domains promote teamwork to build holistic support to respond to comfort care needs.

In this way, health professionals, specifically nurses, must understand this search as a specific need for comfort in a particular and unique area of life, such as survival, accompaniment, and comforting intervention, assuming holistic care in its comprehensive and total concept.

## Figures and Tables

**Table 1:** Participant characterization: diagnosis, age group and sex.

Diagnosis/Age Group	Female	Male	Total
Acute Lymphoblastic Leukemia	2	3	5
30–39		1	1
40–49	2		2
50–59		1	1
60–69		1	1
Acute Myeloid Leukemia	4	2	6
30–39	1		1
40–49	3	1	4
50–59		1	1
Chronic Myeloid Leukemia		1	1
50–59		1	1
Hodgkin Disease	2		2
40–49	2		2
MonoMAC Syndrome	1		1
50–59	1		1
Myelodysplastic Syndrome		1	1
40–49		1	1
Non-Hodgkin Lymphoma	2	2	4
40–49	2	1	3
60–69		1	1
Total	11	9	20

**Table 2:** The presence of GVHD percentage.

Presence of GVHD	Percentage
No	25%
Yes	75%

**Table 3:** Participants number of years after ASCT.

Years After ASCT	Number of Survivors
1	2
2	5
3	2
4	3
5	4
8	1
9	1
10	1
12	1
Total	20

## Data Availability

The data presented in this study are available on request from the corresponding author. The data are not publicly available due to ethical reasons.
